# Navigating Environmental Transitions: the Role of Phenotypic Variation in Bacterial Responses

**DOI:** 10.1128/mbio.02212-22

**Published:** 2022-10-19

**Authors:** Madison R. Spratt, Keara Lane

**Affiliations:** a Department of Molecular Biosciences, Northwestern University, Evanston, Illinois, USA; Boston University; Ohio State University

**Keywords:** phenotypic heterogeneity, environment, stochasticity, dynamics, single cell, bacterial communities, virulence

## Abstract

The ability of bacteria to respond to changes in their environment is critical to their survival, allowing them to withstand stress, form complex communities, and induce virulence responses during host infection. A remarkable feature of many of these bacterial responses is that they are often variable across individual cells, despite occurring in an isogenic population exposed to a homogeneous environmental change, a phenomenon known as phenotypic heterogeneity. Phenotypic heterogeneity can enable bet-hedging or division of labor strategies that allow bacteria to survive fluctuating conditions. Investigating the significance of phenotypic heterogeneity in environmental transitions requires dynamic, single-cell data. Technical advances in quantitative single-cell measurements, imaging, and microfluidics have led to a surge of publications on this topic. Here, we review recent discoveries on single-cell bacterial responses to environmental transitions of various origins and complexities, from simple diauxic shifts to community behaviors in biofilm formation to virulence regulation during infection. We describe how these studies firmly establish that this form of heterogeneity is prevalent and a conserved mechanism by which bacteria cope with fluctuating conditions. We end with an outline of current challenges and future directions for the field. While it remains challenging to predict how an individual bacterium will respond to a given environmental input, we anticipate that capturing the dynamics of the process will begin to resolve this and facilitate rational perturbation of environmental responses for therapeutic and bioengineering purposes.

## INTRODUCTION

**FIG 1:**
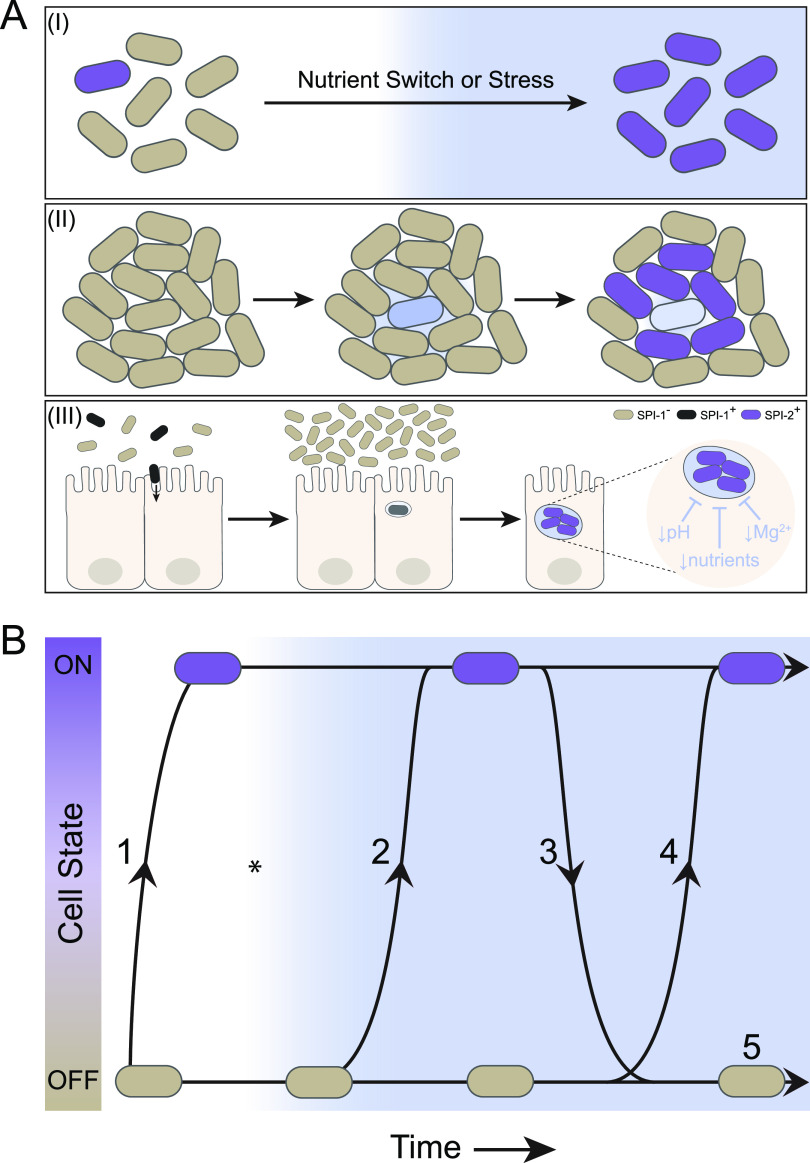
Heterogeneity and dynamics impact how bacteria respond to environmental transitions. (A) Fluctuating environments are a feature of bacteria across biological systems. (I) The local environment of planktonic bacteria can fluctuate, for example, upon a switch in nutrients or the addition of a stress (blue). Independent of the environment, a small fraction (purple) of the clonal population of bacteria express the molecule needed to respond to this change and are better prepared to survive and proliferate when the switch occurs. (II) In a biofilm, environmental fluctuations can occur due to the actions of the population. One bacterium (blue) produces a toxic metabolite. To survive, the neighbors produce a detoxification protein (purple) that reduces the concentration of the metabolite and ensures the population’s survival. (III) During an infection, bacterial pathogens experience environmental fluctuations due to changes in their location and the actions of the host. Phenotypic heterogeneity in the SPI-1 virulence regulon enables Salmonella Typhimurium to survive such transitions. SPI-1**^+^** cells (black) invade the epithelium and promote inflammation, allowing SPI-1^−^ cells (beige) in the gut lumen to proliferate. Once inside the host cell phagosome, the pathogen must contend with additional environmental changes driven by the host cell (blue: decrease in pH, magnesium ions, or nutrients). To survive in the phagosome, precise regulation of virulence dynamics is needed; the pathogen turns off SPI-1 and switches on a second virulence regulon, SPI-2. (B) Dynamic data are necessary to determine the mechanisms underlying bacterial responses to fluctuating environments. There are many ways for a bacterium to transition from an OFF to an ON state; therefore, the response (beige to purple) of a single bacterium to an environmental transition (blue, asterisk) is challenging to predict if only the initial and final states are known. A cell can turn ON before the environmental switch occurs (1), shortly after the switch occurs (2), oscillate between OFF and ON states (3), activate with an additional lag period (4), or never switch to the ON state (5). Following the same cell over time can resolve these ambiguities in how a cell responds to environmental shifts.

Bacteria exist in a world in flux, frequently encountering changes in their surroundings. These changes can range in their origin and complexity, from fluctuations as simple as a resource switch in a planktonic cell’s surroundings to multiplexed host immune responses against an invading pathogenic bacterium (Fig. 1A). In each context, bacteria must adequately sense, process, and invoke functional responses to such changes to ensure their survival and proliferation, and they have evolved strategies to do so even in complex, dynamic environments. Classic studies used population-level techniques and targeted mutations to characterize the molecular basis for bacterial responses to a wide variety of stimuli, revealing key regulatory features of environmental sensing and response (1, 2). While the molecular basis for many bacterial responses has been extensively mapped, it remains challenging to predict how a single cell will respond to a given environmental input, as the response of individuals in a clonal population can vary dramatically despite being genetically identical and residing in a homogeneous environment. This nongenetic variability across individuals is referred to broadly as phenotypic heterogeneity and has been shown to play a significant role in how bacteria respond to environmental transitions.

The stochastic nature of gene expression, commonly referred to as noise, has been well established as the basis from which phenotypic heterogeneity arises (3, 4). Many early observations of phenotypic heterogeneity, including in enzyme synthesis (5), antibiotic susceptibility (6), and chemotaxis (7), attributed the distinct responses of bacteria to the random distribution of low numbers of molecules and, in some cases, used mathematical models to support this claim. However, direct measurements of these distributions had to wait for advances in fluorescent proteins and *in situ* hybridization techniques with single-mRNA resolution. Elegant experiments using fluorescent protein reporters to directly monitor variances in gene transcription and translation validated the early mathematical models, providing experimental evidence for the origins of noise in gene expression (8, 9). Noise can originate from stochasticity associated with the transcription and translation of a gene or random distributions of core cellular components, such as RNA polymerase or ribosomes (3, 4, 10). It has become apparent that bacterial regulatory networks have evolved to propagate and amplify noise to produce functionally distinct subpopulations (11–13). These phenotypically distinct subpopulations can benefit the population as a whole, particularly in instances in which environmental conditions fluctuate (14). This population-level benefit may occur through two potential mechanisms: bet hedging and division of labor. Bet hedging refers to an instance in which a subpopulation is better equipped for survival in the event of an environmental switch at the cost of their proliferation in the current environment (15–18). Several evolutionary criteria must also be met to satisfy a definition of bet hedging. The fitness of the heterogeneous population should exceed that of a homogeneous population under fluctuating conditions, and the frequency of environmental fluctuations should determine the switching rates between subpopulations (16–19). In a division of labor strategy, energetically costly functions required of the population are distributed among discrete coexisting subpopulations, minimizing the burden placed on individuals (20, 21). A key feature of division of labor is that the tasks carried out by each subpopulation are complementary. Unlike in bet hedging where the relative fitness of each subpopulation varies according to the environment, in division of labor the phenotypically distinct subpopulations synergize, and fitness is greatest when they coexist. While it can be challenging to experimentally meet these evolutionary criteria, phenotypic heterogeneity has been associated with growth, stress tolerance, and survival in a range of bacterial species during environmental transitions.

Making connections between phenotypic heterogeneity and its ultimate functional consequences is not trivial, despite its prevalence in bacterial systems. One reason for this is that for a given cell, many possible trajectories can connect the initial and final cell states (Fig. 1B), and neither population average data nor discrete single-cell time point data can resolve these ambiguities. Instead, to begin to determine how phenotypic heterogeneity is established, how it is regulated, and how it impacts cell fate, the response of hundreds, if not thousands, of individual bacteria needs to be followed over the duration of an environmental transition. Microfluidic devices, such as the mother machine and the dual-input mother machine (DIMM), allow bacteria to be continuously imaged over hundreds of generations while also providing precise temporal control of the environment (22–24). Deep learning approaches now allow rapid analysis of the large data sets generated using such devices (25–27). While these approaches are critical to observe and quantify phenotypic heterogeneity in bacteria, they also provide opportunities to gain mechanistic insight into how dynamic behaviors can emerge through the perturbation of well-characterized regulatory networks (28).

The convergence of these technical advances and their application to the study of bacterial responses has led to a rapidly growing understanding of the intricacies of single-cell decision making during environmental transitions. These include broadly applicable principles of growth rate effects on network response (29), visualization of dynamic stochastic events (28, 30, 31), and the ability to link functional significance with observed phenotypic heterogeneity before, during, and after a response to an environmental switch (31–33). Here, we review the significance of phenotypic heterogeneity in bacterial environmental responses and how the application of dynamic, single-cell imaging has begun to allow the causes, regulation, and consequences of this variation to be established. While we have chosen to limit our review to bacterial systems, we note that phenotypic heterogeneity has been extensively studied in other microbes, and many of the concepts described here are likely to be broadly applicable (18, 34–39). Here, we summarize recent work that has established a guiding set of principles that drive single-cell bacterial responses and discuss their implications in environmental transitions that range from simple diauxic shifts to community behaviors in biofilm formation and virulence regulation during infection (Fig. 1A). We emphasize that this is a relatively new field of study and outline current challenges and future areas of interest. Given the wealth of foundational knowledge regarding the molecular mechanisms of bacterial response systems and the rapidly improving technologies to study them with single-cell, temporal resolution, we anticipate the next decade will reveal exciting insights into how bacteria make decisions in fluctuating environments.

## PROCESSING OF ENVIRONMENTAL SIGNALS AT THE SCALE OF INDIVIDUAL BACTERIA

**FIG 2:**
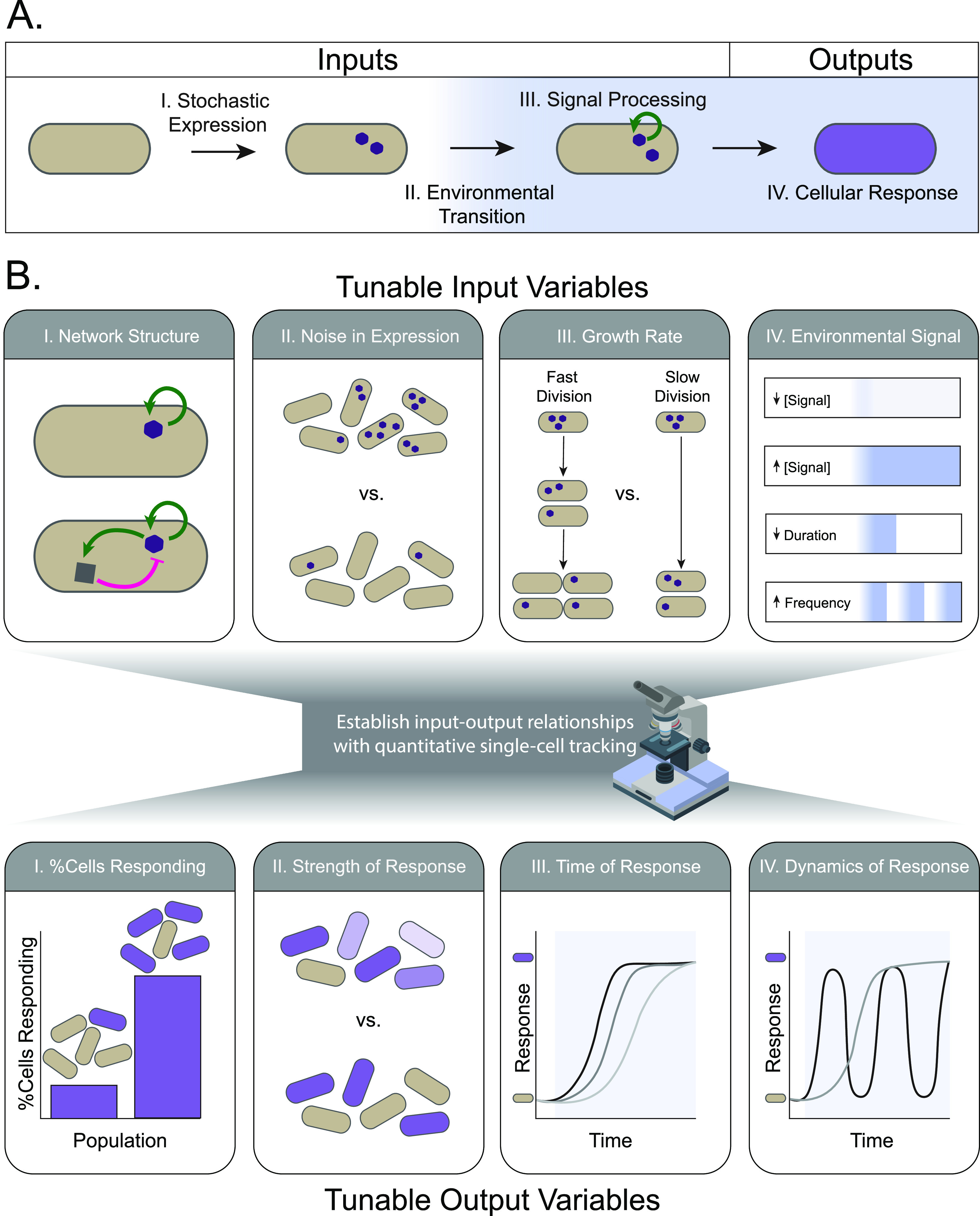
Live-cell dynamic approaches are needed to link input variables to response outputs during environmental transitions. (A) A generalized schematic for a simple, functional response (beige to purple) to an environmental shift (white to blue) is shown. A bacterium in an uninduced environment has stochastic noise in gene expression (I) that produces a molecule (purple hexagon) that will allow a functional response to an environmental shift. Upon sensing an environmental shift (II), the response molecule initiates signal processing (III) that transitions the cell into a functionally responsive state (IV). (B) Tunable input and output variables are displayed for the simple, functional response shown in panel A. Inputs that can determine how single cells will respond to environmental shifts are described from left to right. (I) The structure of the regulatory network downstream of the response molecule determines the functional output; in this example, a network consisting of a simple autoregulatory feedback loop (top) will produce a different response from one with an added indirect negative feedback loop (bottom). (II) Stochastic noise in gene expression can establish cells with various levels of the response molecule prior to the environmental switch. Higher levels of noise (top) create a broader distribution in the population prior to the switch. (III) Growth and division of the bacterium dilute the response molecule prior to the environmental switch. A high growth rate (left) decreases the concentration of response molecules in each cell relative to a lower growth rate (right). (IV) The environmental signal itself can vary the response through its concentration, duration, or frequency. Establishing relationships between the inputs and outputs of a bacterial response requires single-cell resolution over the duration of the environmental transition. Outputs that can be altered as a function of these four inputs include the following from left to right: (I) the proportion of cells that will respond to a given stimulus; (II) the strength of response at the individual level, for example, a graded response in which cells vary incrementally between the beige and purple states (top) or a binary response in which cells are either ON or OFF (bottom); (III) the timing of the response, such that individual cells may transition from the OFF to ON states rapidly (black line) or have a delay following the environmental stimulus (gray lines); and (IV) the dynamics of the response, in that a cell may oscillate between the OFF and ON states following the stimulus (black) or remain in the ON state (gray).

Recent single-cell quantitative analyses have revealed principles of bacterial responses during simple, single environmental switches. Here, we focus on framing these principles as part of an input-output relationship for dynamic, single-cell responses (Fig. 2). The structure of a gene regulatory network is essential to these relationships, allowing for the amplification of noise and enabling genetically identical cells to stochastically switch into distinct cell states. The effects of such network structures have been well resolved through biochemical and genetic approaches and have been reviewed elsewhere (11, 13, 40, 41). A thorough characterization of gene regulatory networks provides the context needed to understand how other inputs, including noise, growth rate, and features of the environmental signal itself, are integrated to produce a response. Linking these three input features of a bacterial response with a consequential response output requires that the cells be tracked over the course of an environmental fluctuation. Below, we discuss instances in which inputs have been successfully coupled to response outputs through both experimental and modeling approaches.

### Noise in expression establishes heterogeneity prior to an input signal.

Extensive studies have established how noise in gene expression and its effects at the phenotypic level are genetically encoded (3, 4, 42). However, the levels of noise for a given gene and its contribution to an environmental response remain unresolved in most bacterial systems. It was recently shown that noise is also context dependent and varies globally with growth rate and stress conditions (43). Thus, direct measurements of noise under the conditions of interest are needed to establish its connection to environmental response outcome.

An open question in many bacterial response systems is how small of a stochastic difference in gene expression can induce differential responses. While the use of fluorescent reporters in combination with a microfluidic device can provide critical dynamic data regarding expression outcomes of single bacteria following an environmental shift, this approach is not sensitive enough to resolve the lower limit of variation in gene expression prior to the response occurring. Recent work has addressed this in the *lac* operon response, where it had previously been shown that responsive and unresponsive subpopulations form following a carbon source switch (44, 45). Combining a dynamic, microfluidics-based approach with fluorescence lifetime correlation spectroscopy (46) revealed that a stochastic difference on the order of a single molecule prior to a carbon source switch drives the formation of these two subpopulations following the switch (47). The responsive population undergoes a rapid response to the transition, inducing a positive feedback loop that activates *lacZ* expression at high levels. The unresponsive population has a delayed response, as it requires a stochastic expression event prior to the activation of the feedback loop, illustrating an instance in which the level of noise directly correlates with a difference in the timing of the response in single cells (47).

Noise is inherently an unstable characteristic of single cells, and recent work has established rich dynamic regulation underlying stochastic events prior to an environmental switch. Pulsatile signaling dynamics are prevalent across biological systems, and transient pulsing may enable bet hedging (48, 49). One demonstration of this is the expression of the alternative sigma factor RpoS in Escherichia coli, a key regulator of stress responses (33, 50, 51). Monitoring the activity of RpoS in unstressed conditions in a mother machine revealed that subpopulations expressing RpoS are established through pulses of expression, preparing a subpopulation for an exposure to stress without a long-term commitment to a phenotype (33). Another study has similarly quantified the expression dynamics of a wide range of stress response regulators in E. coli using a mother machine (31). Here, it was found that stochastic pulsing under unstressed conditions is a feature of virtually all stress response regulators surveyed and that pulse characteristics are distinct for each regulator (31). Pulsatile dynamics have also been observed in other well-studied bacterial transcriptional cascades, including those involved in flagellum synthesis (28) and competence responses (52), implying that stochastic pulses of expression may be a prevalent feature of bacterial gene regulatory circuits and a common mechanism that establishes heterogeneity prior to an environmental switch.

### Growth rate can determine the sensitivity of a bacterial response.

An underlying caveat to the degree to which gene expression noise can affect bacterial responses is the rapid rate at which bacteria divide. Cellular properties such as ribosomal content and metabolism are globally affected by growth rate, and it has been established that growth rate-dependent shifts in resources such as ribosomes and RNA polymerase are coupled with predictable changes in transcription and translation (53–55). More recently, modeling work has addressed how growth rate can affect the sensitivity of environmental responses (Fig. 2B), revealing that increased division rates dilute regulatory network components, transcripts, environmental signals, and ligands such that increasing the growth rate will always decrease sensitivity to an environmental signal (29). Notably, this provides a simple mechanism by which cells may mute environmental responses during rapid growth and conversely become hypersensitive to the same signals when conditions are less than optimal. These models were validated through experiments again investigating the *lac* operon, in which it was found that growth-arrested cells were indeed more sensitive to carbon source transitions and activated the *lac* operon at higher rates than cells growing more rapidly (29). While mathematically growth rate affects virtually all bacterial regulatory systems through dilution and effects on global noise levels, it remains to be seen in which instances these effects play a significant role in determining response under physiologically relevant conditions.

### Dynamic features of environmental signals impact bacterial outcomes.

The response of a bacterial cell to an environmental signal is unsurprisingly dependent on the signal itself, in particular its strength or duration (Fig. 2B). Less obvious, however, is the extent to which dynamic features of an environmental signal can change bacterial responses. In Bacillus subtilis, the rate at which an environmental stress is applied to cells results in distinct responses (56). Step changes in a stress signal induce both a general and a stress-specific response, while ramp changes induce only a stress-specific response (56). Similar responses to the rate of environmental change have been found to occur in other organisms (56–58). While dynamic features of environmental signals, such as the frequency of the input or the rate of change, are difficult to measure in natural systems, microfluidic devices can be used to determine whether individual bacteria can process temporal patterns in the input signal in different ways (23). The capacity of bacteria to decode such patterns could be a strategy to filter fluctuations in environmental signals and avoid activation of a response to a transient signal.

### Single-cell processing in complex environments.

While the study of simple, single environmental condition changes has established general principles of bacterial information processing, bacteria frequently encounter far more complex environmental transitions that require them to process multiple concurrent signals, make decisions in the presence of conflicting signals, or prepare for future environmental changes. To address the first of these challenges, regulatory networks are often evolved to integrate multiple environmental signals to converge on the same functional output. Integration can occur at a variety of points in the regulatory network, including the sensing of the signals (59) or the response processing (60), and can be additive, synergistic, or redundant depending on the mechanism by which they are integrated. As such, assessing single-cell responses to combinations of signals can provide insight into how responses are regulated under physiologically relevant conditions.

Bacteria may also encounter environmental signals that drive opposing outcomes, and investigating how they resolve such conflicts can reveal logistical frameworks of bacterial decision making. One well-studied model of decision making in bacteria is that of sporulation and competence in B. subtilis. When stressed, B. subtilis cells enter a state in which they will commit to either a dormant, stress-resistant spore phenotype or a competent cell phenotype capable of DNA uptake (61). Live-cell imaging revealed that commitment is achieved through a molecular “race” in which the two signaling pathways are induced in parallel, and the cell ultimately commits to the phenotype that reaches a commitment point more rapidly (62). Similar strategies are used to resolve signaling conflicts in other bacterial species. In the cyanobacterium Crocosphaera watsonii, the processes of nitrogen fixation and photosynthesis are temporally segregated by night and day (63). When cells are exposed to light during night hours, individual cells resolve this conflict by committing to either photosynthesis or nitrogen fixation. This reveals a strategy in which subpopulations are specialized to perform both functions during times of day-night transition without requiring the greater energetic costs of individuals performing both functions (63). In E. coli, the chemotaxis response to conflicting signals has distinct commitment strategies dependent on the signals sensed (64). Indole and low-pH signals equally repel the migration of cells on their own. When low pH is combined with an amino acid attractant, cells are similarly uniformly repelled. However, when an indole is combined with the same attractant, a subpopulation of cells migrates toward the attractant, suggesting a bet-hedging-like strategy for response to this specific combination of cues (64). This instance illustrates that while cellular decision making can be as simple as a commitment to a dominant signal, context is critical, and elucidating the logic of bacterial responses requires single-cell resolution.

Environments that bacteria encounter are complex not just in their composition but also in the temporal order in which signals appear, requiring regulation strategies in which a response to one signal alters the response to a subsequent signal. This is a common feature of stress response systems, often referred to as cross-protection, and has been demonstrated at the population level across antibiotic (65), osmotic (66), bile (67), acid (68), and starvation (66, 69) responses. Recent work has demonstrated that noise in gene expression may determine the cross-protective response at the level of individual cells, as is the case with the acid response operon *gadBC* in E. coli following exposure to antibiotics (70). Cross-protective responses can also activate independent pathways in a temporally structured, anticipatory manner. This is the case in E. coli temperature sensing, in which the temperature sensing response has evolved such that an increase in temperature initiates a response to prepare the cell for an impending drop in oxygen, as occurs upon entry into the gut (71). Anticipatory responses have been shown to be evolutionarily favorable (72); however, their prevalence in other bacteria and how homogenous they are across clonal populations remain to be seen.

## PROCESSING OF ENVIRONMENTAL SIGNALS AT THE SCALE OF BACTERIAL COMMUNITIES

Bacteria do not live in isolation, and the environment an individual bacterium experiences is often a function of the larger population within its vicinity (Fig. 1A). As such, differential responses of individuals can be driven by the state and interactions between and within a bacterial population. A fundamental example of this is entry into the stationary growth phase. As a population of bacteria increases in density and depletes local resources, cells must activate a transcriptional response to slow growth and tolerate the increasingly nutrient-poor surroundings (50, 51). Activation of this response is heterogeneous in E. coli, with both cell size and the time of entry into stationary phase inversely correlated with the time of exit (73, 74). Variation in lag time is associated with tolerance to antibiotics and may be a strategy to maintain a stress-resistant population over the course of this environmental transition (73, 75, 76). This represents an instance in which environmental conditions produced by the population initiate differential responses of individuals with functional advantages.

While perhaps the best studied, stationary-phase entry is not the only stage of population growth that promotes heterogeneous bacterial responses. In B. subtilis and E. coli, for example, it has been well established that acetate is produced during aerobic growth to levels that become toxic to the population. This is followed by a detoxification response, in which acetate is metabolized to a neutral metabolite, acetoin (77, 78). Despite this pathway being well established from a biochemical and genetic perspective, single-cell analysis of this environmental transition in B. subtilis revealed that discrete subpopulations produced or consumed acetate and cells expressing genes required to metabolize acetate grow slower than their nonmetabolizing counterparts (79). However, the presence of acetate detoxifiers substantially increased the growth of a mutant population unable to perform this function, demonstrating a tradeoff between the costs of acetate consumption on the individual and the benefit to the population.

### Heterogeneity in quorum sensing.

In many cases where the environment changes as a consequence of the actions of the population, each cell responds as an individual, as outlined above. However, in other cases, the population of cells can instead respond synchronously in a phenomenon known as quorum sensing (80–83). Certain bacterial responses, including competence, bioluminescence, and virulence factor expression, may be more effective if induced in a coordinated manner when bacterial cell density is high (84, 85). To achieve this, bacteria secrete cell-to-cell signaling molecules known as autoinducers, which allow bacteria to induce a coordinated response at a particular cell density threshold. As the basis of quorum sensing suggests advantages to coordinated population behaviors, responses to environmental transitions involving quorum sensing were thought to be homogeneous. However, most quorum-sensing studies use bulk culture measurements, potentially masking underlying phenotypic heterogeneity (86–88).

In many cases, examining quorum sensing at the single-cell level revealed two coexisting subpopulations, quorum-sensing responders and nonresponders (89, 90). This heterogeneity is reversible as each subpopulation can recreate the parental response distributions. This phenotypic heterogeneity is either autoinducer dependent, indicating that subsaturating levels of the molecule are responsible for the heterogeneity (90), or autoinducer independent (89). Gene expression variation has been identified at multiple levels of the network, from the autoinducer synthase to the receptor and secreted product (91–93). While noise in gene expression may be responsible, the structure of quorum-sensing regulatory networks and physiological effects of autoinducer secretion may also contribute to heterogeneity. A recent modeling approach involving the monostable production of autoinducers along with a fitness cost associated with autoinducer production was able to simulate phenotypic heterogeneity (94). While the model remains to be tested experimentally, it suggests an alternative molecular mechanism that can lead to heterogeneous upregulation of autoinducer expression in single cells.

Two recent studies have attempted to determine the single-cell responses underlying heterogeneity in quorum sensing. Individual cells could respond to increasing concentrations of autoinducer in the environment in either a digital or an analog manner. The expression of *lasB*, a quorum-sensing target gene in the pathogen Pseudomonas aeruginosa, shows a graded, linear response at the population level to increasing cell density (95). However, at the single-cell level, the response is bimodal. As cell density changes, so too does the fraction of cells induced and the intensity of induction, indicating that the response is a mixture of digital and analog features. Although heterogeneity is now well documented for many quorum-sensing genes, the dynamics underlying this variation are less well characterized. In Sinorhizobium meliloti, the autoinducer synthase gene *sinI* is heterogeneously expressed (96). Dynamic data determined that *sinI* is expressed in a pulsatile manner, that pulse frequency can be tuned by environmental cues, such as phosphate starvation, and that frequency regulates the timing of quorum-sensing responses. Together, these studies suggest that quorum sensing is rich in dynamic behavior and that bacteria may be able to fine-tune the quorum-sensing response to small changes in cell density. Heterogeneity in quorum-sensing systems can have functional consequences for bacterial communities, and this has primarily been demonstrated for stages of biofilm formation (90, 97). The growing prevalence of heterogeneity in quorum-sensing systems and the tunability of these responses suggest that variation may be an inherent feature of these regulatory networks and may play a critical role in how bacteria regulate collective decision making in response to environmental changes.

### Biofilms and dynamic cell responses.

As the level of interaction and organization within a bacterial community increases, so does phenotypic diversity. Biofilms are complex, spatially structured communities of bacteria encased in a matrix of polysaccharides that can form in response to stressful environmental conditions. While spatial gradients in nutrients and chemicals can elicit heterogeneity in gene expression across the biofilm, here we focus on recent studies connecting the dynamics of cell signaling molecules with environmental sensing and decision making in a biofilm (98–101).

Although heterogeneity in cell decision making is well documented in planktonic culture (61–63), the extent to which similar responses occur in bacterial communities, such as biofilms, is not well understood. One example where cells must commit to one of two mutually exclusive lifestyle choices is the transition from a planktonic to a biofilm state. This transition is generally associated with the downregulation of motility genes and the upregulation of matrix production genes (102, 103). In P. aeruginosa, the initial surface-sensing and microcolony-forming stage of biofilm formation is associated with an increase in the levels of the second messenger, cyclic di-GMP (c-di-GMP) (104, 105). However, c-di-GMP induction is not homogeneous. A fluorescent reporter for c-di-GMP revealed the presence of two functionally distinct subpopulations: cells with high c-di-GMP rapidly initiate matrix production, while those with low levels continue to survey the surface (106). This study suggests that commitment to a biofilm fate is heterogeneous, and whether these subpopulations reflect a bet-hedging or division of labor strategy warrants further investigation.

Pulsatile responses can generate transient heterogeneity in a bacterial population and allow incompatible cell states to coexist (48, 49, 63). In B. subtilis, stochastic pulsing of the sigma factor σ^B^ is associated with stress responses in planktonic culture (30). However, the extent to which spatial heterogeneity in σ^B^ in biofilms may originate from stochastic pulsing was unknown. Using time-lapse microscopy, σ^B^ was found to be heterogeneously expressed in a gradient within the biofilm (107). Furthermore, a combination of modeling and experiments revealed that pulsatile σ^B^ expression allows spore-forming cells to coexist in the same spatial location as cells expressing σ^B^, despite σ^B^ acting as a repressor of spore formation. Oscillations can occur not only at the level of individual cells but also at the scale of the entire biofilm. Oscillations in biofilm growth arise due to metabolic codependence between cells at the interior and those at the periphery and increase the capacity of the biofilm to withstand stress (108). These temporal patterns may be an additional regulatory strategy to enable individuals within a biofilm to respond at a population level and survive environmental stress.

## PROCESSING OF ENVIRONMENTAL SIGNALS IN THE CONTEXT OF HOST-PATHOGEN INTERACTIONS

Stable environmental conditions rarely feature in the life cycle of a bacterial pathogen. Instead, from entry into a host to colonization and eventual transmission, bacterial pathogens transit through several dynamic and heterogeneous environments, many of which present unique navigational challenges. For instance, pathogens must sense and respond to environmental shifts within a limited time window to avoid neutralization by a host and endure environments that are in continuous flux as both host and pathogen attempt to modify them in their favor. Despite the additional complexity associated with these environmental transitions, the fundamental challenge remains the same: how does a pathogen contend with and survive fluctuating host environments?

Elegant genetic and biochemical studies have characterized the molecular mechanisms that pathogens use to respond to changes in the host environment and to induce virulence genes needed for colonization and proliferation within a host (109–111). Virulence gene expression is temporally and spatially regulated (112, 113); misexpression of these genes can compromise bacterial survival due to metabolic costs or premature activation of the host immune response. However, how an individual pathogen accomplishes this feat and optimizes virulence gene expression is less clear, especially given the challenges outlined above. Phenotypic heterogeneity may be one approach pathogens use to reconcile the need for a rapid and reliable response to an environmental shift with the cost of virulence gene expression. However, while *in vitro* studies on phenotypic heterogeneity in nonpathogenic strains have flourished in recent years (28, 31, 33, 47), there has not been a corresponding increase in applying these approaches or concepts to an infection context. In this section, we review pioneering studies on the role of phenotypic heterogeneity in virulence gene expression and highlight several recent studies that implicate it more broadly in bacterial pathogenesis. These studies have overcome the challenge of directly linking input signals with output responses *in vivo* by combining *in vitro* microfluidic approaches, similar to those discussed earlier, and mathematical modeling to determine the dynamics of the system and generate predictions that can then be tested *in vivo*. Although persisters are relevant to infection and phenotypic heterogeneity, we have chosen not to include them here due to several recent reviews on the topic (114–117).

### Division of labor and heterogeneity in virulence gene expression.

The enteric pathogen Salmonella enterica serovar Typhimurium has two primary virulence regulons, Salmonella pathogenicity islands 1 (SPI-1) and 2 (SPI-2), each of which encodes a type three secretion system (T3SS) (118). Pathogen survival is tightly linked to virulence gene expression. Therefore, a surprising finding was that SPI-1 is heterogeneously expressed after exposure to a uniform stimulus (119). In the gut lumen, the SPI-1^+^ fraction of the population invades the intestinal epithelium, and the resulting inflammation allows the SPI-1^−^ cells to proliferate at the expense of the microbiota (Fig. 1A) (120). Both subpopulations are needed for a productive infection; modeling and *in vivo* experiments determined that perturbing the ratio of SPI-1^−^ to SPI-1^+^ cells allows avirulent cheaters to overtake the wild-type *S.* Typhimurium, and the host to clear the infection (121). In addition to this division of labor, heterogeneity in SPI-1 may also be a bet-hedging strategy. SPI-1^+^ cells have a reduced growth rate *in vitro* compared to SPI-1^−^ cells but survive antibiotic treatment at higher rates (32, 122). Although the functional consequences of SPI-1 heterogeneity for infection are well established, due to the complexity of the host environment and technical challenges associated with *in vivo* measurements, less is known about the extent to which input variables can tune the response (Fig. 2B). Several environmental inputs (high salt and low oxygen tension) induce heterogeneous SPI-1 expression (122, 123). Short-chain fatty acids (SCFAs) produced by gut microbes reduce SPI-1 expression at the population level and impair invasion, but the underlying mechanism was unknown (124, 125). By combining SPI-1 fluorescent reporter strains, microfluidics, and modeling, SCFAs were found to reduce the growth rate of SPI-1^+^ cells and, as a result, tune the fraction of SPI-1^+^ cells rather than SPI-1 expression levels in single cells (126). Heterogeneity in virulence expression has also been noted for RovA, a virulence regulator in Yersinia pseudotuberculosis (127). RovA was known to respond to temperature changes upon host entry, but single-cell analysis revealed that this response is bistable and can be tuned by nutrient availability. Perturbing the ratio of RovA^+^ to RovA^−^ cells alters tissue colonization and disease severity, confirming a functional role for RovA heterogeneity *in vivo*. These examples, along with several others (128–130), highlight the significance of heterogeneity in virulence gene expression during infection and demonstrate how iterating between *in vitro* approaches, mathematical modeling, and *in vivo* infection models can lead to mechanistic insight.

### Heterogeneity in virulence gene downregulation.

Ensuring virulence genes switch off when pathogens transition into noninducing environments is as critical for pathogen survival as their appropriate induction. However, the strategies pathogens use to avoid premature repression of virulence genes are relatively understudied at the single-cell level compared to those involved in induction. Several single-cell studies suggest that heterogeneity in virulence gene downregulation may be a common feature of the transition out of an inducing environment. ScanLag, an automated approach to extract lag and doubling times, was used to identify bimodality in enteropathogenic E. coli colony appearance following an environmental switch out of virulence-activating conditions (131, 132). Smaller colonies retain expression of the *per* virulence operon over multiple generations, and this bimodality is regulated by a hysteretic switch. Multigeneration stability of virulence gene expression under noninducing conditions has also been observed for a toxin-coregulated pilus gene in Vibrio cholerae and contrasts with the rapid downregulation of virulence genes observed in other pathogens (133, 134). Maintaining virulence gene expression in a fraction of cells may be a strategy that is used to mitigate the uncertainty associated with entry into a noninducing environment.

Virulence-repressing environments can be long lasting or transient, and the mechanisms pathogens use to distinguish these conditions and cycle between virulence-inducing and -repressing conditions are not well known. For example, following the invasion of host epithelial cells, *S.* Typhimurium rapidly represses SPI-1 and switches on SPI-2 in the phagosome (135). Heterogeneous reexpression of SPI-1, linked to Akt survival signaling, occurs when the pathogen later exits the phagosome and enters the host cytoplasm (136). Cross-regulation between the two virulence regulons is involved in this virulence switching behavior, but how the pathogen balances the competing needs of rapid switching and tight repression under long-lived noninducing conditions remains unclear, as does the functional role of heterogeneous SPI-1 activation in the cytoplasm (137–139).

### Spatially regulated heterogeneity in virulence gene expression.

Several environmental transitions during infection pose an additional challenge as they isolate individual bacteria, thus forcing a community of bacteria to be reestablished from a single cell. This can occur, for instance, upon seeding a systemic tissue site or after the entry of a single bacterium into a phagosome. Several studies have begun to examine the extent and functional relevance of phenotypic heterogeneity in these newly formed communities. For example, when individual Mycobacterium tuberculosis lesions in the mouse lung were examined with single-pathogen resolution, spatial heterogeneity in pH and chloride environments was observed, along with variation in replication status and antibiotic sensitivity (140). While the functional relevance of this variation requires further study, phenotypic heterogeneity in a bacterial community such as this could potentially be a bet-hedging strategy to ensure survival, or it may allow members of the bacterial community to distribute tasks and cooperate to withstand the host immune response. Environmental cues from host immune cells may also drive phenotypic heterogeneity in a pathogen. Y. pseudotuberculosis forms microcolonies in the spleen of infected mice, but the extent to which environmental signals from the host impact microcolony structure or heterogeneity was unknown. With the use of a fluorescent reporter that responds to nitric oxide (NO), bacteria on the periphery of a colony were found to express a NO-detoxifying enzyme to limit NO diffusion and protect cells at the center of the colony (141). Evidence that these subpopulations are functionally important and may cooperate came from deletion experiments that prevented detoxification of NO in the periphery and reduced the fitness of the mutant colony. Determining how phenotypic heterogeneity is impacted by tissue context is challenging as it requires techniques that preserve positional information to link it to pathogen variation. This type of information is not captured by *in vitro* systems, such as the mother machine, or single-cell profiling methods that dissociate cells in a tissue, such as single-cell RNA-Seq. As such, *ex vivo* systems that are amenable to perturbations and imaging, such as organoids or droplet microfluidic culture systems, will be instrumental in determining the regulatory mechanisms underlying community reestablishment from single bacteria after environmental transitions (142–144).

## CONCLUDING REMARKS AND FUTURE PERSPECTIVES

Environmental transitions are a universal feature of the life cycle of a bacterium. Trajectories of single-cell responses during environmental transitions enable a direct, unambiguous connection between the initial state and final cell decisions that are made, be they growth, differentiation, or survival (Fig. 1B). In addition, the extent to which input variables, such as noise, growth rate, or duration of the signal, can tune distinct features of the output, such as the fraction of responders or time to respond, can be determined (Fig. 2B). Unraveling the stochastic, dynamic behaviors underlying bacterial decision making may provide opportunities to predict or perturb these decisions in a rational way.

Technical advances in imaging, microfluidics, reporter design, and analysis software have been instrumental in most of the studies outlined here (22–27). The capacity to accurately measure the numbers of molecules inside single bacterial cells continues to increase, and with it emerges new insight into how cells process stimuli and make reliable decisions (29, 47). However, many foundational discoveries on the role of phenotypic heterogeneity have focused on a narrow set of regulatory networks. A challenge for the future will be to assess the extent to which these findings are broadly applicable. This will require technical improvements in throughput, parallelization, and the capacity for multiplexed measurements in single cells. The scope of the field should also be broadened regarding the bacteria studied. Many of the guiding principles in phenotypic heterogeneity have come from *in vitro* studies on model, nonpathogenic bacteria, such as E. coli and B. subtilis. The extent to which these findings will apply to either pathogenic bacteria or natural isolates of bacteria warrants investigation. Due to the diversity in the bacterial world and the myriad types of fluctuating environments bacteria encounter, distinct strategies may have evolved to contend with the vast array of environmental transitions. Therefore, examining environmental transitions in a broader and more diverse range of bacteria is a key next step.

Future work in this area will also benefit from efforts to determine how the context of the natural environment and dynamic features of input signals regulate the bacterial response. During an infection or in a natural environment, unknown constraints or inputs to the system may exist that are difficult, if not impossible, to simulate *in vitro*. Therefore, while *in vitro* results provide essential information about how the regulatory network can work, these results may not reflect how the system functions in a natural setting. Iterating between quantitative, dynamic *in vitro* experiments and *in vivo* infection models to test hypotheses that emerge may be a fruitful approach to resolve this. In addition, environmental changes applied in the lab are likely a poor reflection of those found in natural settings as they rarely incorporate temporal features of the input, such as a ramp change in lieu of a step change, a pulsatile input, or changes in the frequency of the input. Identifying the dynamic features of an input signal that encode information is challenging but necessary to discover how bacteria respond to environmental transitions.
